# Supplementary material for: Functional and cognitive outcomes after COVID-19 delirium
Source: Eur Geriatr Med. 2020 Jul 14;11(5):857–62. doi: 10.1007/s41999-020-00353-8 (PMC7358317; doi:10.1007/s41999-020-00353-8)
Supplement: Supplementary file 1 — Supplementary file1 (DOCX 28 kb) [file 41999_2020_353_MOESM1_ESM.docx]

**Supplementary Table.** 4AT in participants with and without delirium

|  | |  | **No Delirium (%)** | **Delirium (%)** | **P value** |
| --- | --- | --- | --- | --- | --- |
| Alertness | |  |  |  | <0.01 |
|  | 0  4 | | 16 (73%) | 6 (23%) |  |
|  |  |  | 0 (0%) | 8 (100%) |  |
| AMT4 | |  |  |  | <0.01 |
|  | 0  1  2 | | 14 (93%) | 1 (7%) |  |
|  |  |  | 1 (20%) | 4 (80%) |  |
|  |  |  | 1 (10%) | 9 (90%) |  |
| Attention | |  |  |  | <0.01 |
|  | 0  1  2 | | 14 (93%) | 1 (7%) |  |
|  |  |  | 2 (40%) | 3 (60%) |  |
|  |  |  | 0 (0%) | 10 (100%) |  |
| Acute change  or fluctuating  course | |  |  |  | <0.01 |
|  | 0  4 | | 16 (100%) | 0 (0%) |  |
|  |  |  | 0 (0%) | 14 (100%) |  |
